# Patient-reported outcomes in palopegteriparatide-treated adults with hypoparathyroidism: PaTH Forward trial extension

**DOI:** 10.1210/clinem/dgaf653

**Published:** 2025-12-09

**Authors:** Mishaela Rubin, Andrea Palermo, Tamara Vokes, Aliya A Khan, Peter Schwarz, Filomena Cetani, Uberto Pagotto, Elena Tsourdi, Tanja Sikjaer, Kathryn M Pfeiffer, Meryl Brod, Lori D McLeod, Carol Zhao, Wahidullah Noori, Aimee D Shu, Alden R Smith

**Affiliations:** Department of Endocrinology, Columbia University, New York, NY 10032, USA; Research Unit of Metabolic Bone and Thyroid Disorders, Department of Medicine and Surgery, Università Campus Bio-Medico di Roma, 00128 Rome, Italy; Unit of Metabolic Bone and Thyroid Disorders, Fondazione Policlinico Universitario Campus Bio-Medico, 00128 Rome, Italy; Department of Medicine - Endocrinology, The University of Chicago, Chicago, IL 60637, USA; Department of Clinical Medicine, McMaster University, Hamilton, ON L8S 4L8, Canada; Department of Endocrinology, Rigshospitalet and University of Copenhagen, 2100 Copenhagen, Denmark; Department of Clinical and Experimental Medicine, Endocrine Unit, University of Pisa, 56124 Pisa, Italy; Division of Endocrinology and Diabetes Prevention and Care, IRCCS Azienda Ospedaliero-Universitaria di Bologna, Department of Medical and Surgical Sciences, Alma Mater Studiorum University of Bologna, 40100 Bologna BO, Italy; Department of Medicine III and Center for Healthy Aging, Technische Universität Dresden, 01069 Dresden, Germany; Department of Endocrinology and Internal Medicine, Aarhus University Hospital, 8200 Aarhus N, Denmark; The Brod Group, Mill Valley, CA 94941, USA; The Brod Group, Mill Valley, CA 94941, USA; RTI Health Solutions, Research Triangle Park, NC 27709, USA; Endocrine and Rare Disease Medical Sciences, Ascendis Pharma, Inc, Palo Alto, CA 94304, USA; Endocrine and Rare Disease Medical Sciences, Ascendis Pharma, Inc, Palo Alto, CA 94304, USA; Endocrine and Rare Disease Medical Sciences, Ascendis Pharma, Inc, Palo Alto, CA 94304, USA; Endocrine and Rare Disease Medical Sciences, Ascendis Pharma, Inc, Palo Alto, CA 94304, USA

**Keywords:** hypoparathyroidism, quality of life, palopegteriparatide, patient-reported outcomes

## Abstract

**Context:**

Individuals with hypoparathyroidism experience a range of physical and cognitive symptoms and reduced quality of life (QoL) despite management with conventional therapy (active vitamin D and calcium).

**Objective:**

This analysis investigated the long-term impact of parathyroid hormone (PTH) replacement therapy with palopegteriparatide (YORVIPATH®) on symptoms, daily functioning, and well-being in adults with chronic hypoparathyroidism. Associations between patient characteristics and changes in patient-reported outcomes (PROs) were also analyzed.

**Methods:**

PaTH Forward was a phase 2 clinical trial of palopegteriparatide with a 4-week randomized, double-blind, placebo-controlled period followed by an open-label extension period lasting through trial week 266. PRO measures were collected at baseline, weeks 4, 12, 26, 58, and annually thereafter through the end of the trial. The Hypoparathyroidism Patient Experience Scales (HPES) assess disease-specific symptoms and impacts on functioning and well-being. The Short Form Health Survey (SF-36v2) measures general health-related QoL. Data were analyzed using descriptive statistics and Mixed Models for Repeated Measures.

**Results:**

Palopegteriparatide treatment demonstrated significant improvements from baseline in disease-specific symptoms and impacts on daily functioning and well-being at week 12, which were sustained through week 110. Mean changes in PROs met thresholds for clinically meaningful within-patient improvement. Significant improvements in general health-related QoL were also shown in SF-36v2 scores. Results were generally similar across demographics and patient characteristics.

**Conclusion:**

Through week 110 of the PaTH Forward trial, PTH replacement therapy with palopegteriparatide was associated with significant improvements in disease-specific symptoms and impacts on daily functioning and well-being, as well as general health-related QoL.

Hypoparathyroidism is an endocrine disease caused by insufficient levels of parathyroid hormone (PTH), which functions as a key regulator of calcium and phosphate homeostasis by acting directly on bone and kidney and indirectly on the intestine via calcitriol (1). Hypoparathyroidism can impair both physical and cognitive function, and individuals with hypoparathyroidism may experience severe short-term and long-term complications (1-3). Common physical signs and symptoms associated with hypoparathyroidism include fatigue, muscle weakness, muscle cramping, muscle spasms, and tingling or numbness in the extremities or around the mouth and eyes (1). Cognitive symptoms may include problems with memory, concentration, and slow or confused thinking, as well as neuropsychiatric symptoms, including anxiety and depression (1, 4, 5). Conventional therapy for the management of hypoparathyroidism consists of active vitamin D and oral calcium, often at very high doses (4). Serious long-term complications related to hypoparathyroidism managed with conventional therapy include kidney disease, seizures, depression or bipolar affective disorder, cataracts, and ectopic calcifications (1, 6-9).

Conventional therapy aims to alleviate hypocalcemia, but it does not address insufficient PTH levels (10). Research has indicated that even when receiving conventional therapy for hypoparathyroidism, individuals continue to experience a high symptom burden and impairment of functioning and daily life, including interference in work, sleep, physical activity, and relationships (5, 11, 12). A number of studies have shown significantly reduced quality of life (QoL) in individuals with hypoparathyroidism compared to normative reference data on a range of general health-related QoL measures, including the 36-item Short Form Health Survey, version 2 (SF-36v2) and the WHO-5 Well-Being Index, even when treated with conventional therapy (13-15). This suggests a possible direct effect of PTH itself on neurocognitive health, independent of maintenance of normocalcemia and freedom from conventional therapy.

In recent years, guidelines and consensus statements have recognized PTH therapy as a treatment option for adults with hypoparathyroidism who are not adequately controlled by conventional therapy (10, 16). The 2022 Guidelines from the Second International Workshop consider inadequate control to be any one of the following: symptomatic hypocalcemia, hyperphosphatemia, renal insufficiency, hypercalciuria, or poor QoL (16). Palopegteriparatide (TransCon® PTH) is a prodrug of PTH (1-34), administered subcutaneously once daily and designed to provide active PTH within the physiological range for 24 hours/day (17, 18). It consists of PTH (1-34), bound to an inert methoxypolyethylene glycol (mPEG) carrier via a TransCon linker. Upon exposure to physiological pH and temperature, autocleavage of the linker occurs providing predictable sustained release of active PTH (17, 18). The PTH released from palopegteriparatide has an apparent half-life of approximately 60 hours. Palopegteriparatide has received regulatory approval under the brand name YORVIPATH® in the United States, European Union, and other regions (19-22). This design of palopegteriparatide as a PTH replacement therapy provides the physiological effects of endogenous PTH at both the receptor and tissue level.

As reported for the 4- and 26-week double-blind, placebo-controlled periods of PaTH Forward and PaTHway trials, respectively, palopegteriparatide is the first and only PTH replacement therapy for hypoparathyroidism shown to significantly improve well-being and QoL compared to placebo using general SF-36 and disease-specific measures (23, 24). In the phase 2 PaTH Forward trial, treatment with palopegteriparatide was associated with significantly greater improvements from baseline in both the physical and mental component scores of the SF-36v2, and Hypoparathyroidism Patient Experience Scale (HPES) Symptom and Impact domain scores, compared with placebo within 4 weeks of treatment, which were sustained through week 26 of the trial (23). Similar results were recently reported through week 52 of the phase 3 PaTHway trial (25), but the longer-term impacts have not been examined. The purpose of this analysis was to investigate the effects of open-label treatment with palopegteriparatide on disease-specific symptoms and impacts on daily functioning and well-being, as well as general QoL, in adults with chronic hypoparathyroidism through week 110 of the phase 2 PaTH Forward trial. The analysis also explored participant demographic and disease characteristics that might predict improvements in symptoms and impacts with treatment over an extended period to assess whether palopegteriparatide may be particularly beneficial to certain groups.

## Materials and methods

### Trial design

PaTH Forward was a phase 2 clinical trial with a 4-week randomized, double-blind, placebo-controlled period and open-label extension through week 266 designed to investigate the efficacy and safety of palopegteriparatide in adults with chronic hypoparathyroidism. The protocol was reviewed by appropriate institutional review boards and independent ethics committees, and participants provided signed informed consent before enrolling in the trial (ClinicalTrials.gov identifier: NCT04009291; EudraCT No.: 2018-004815-33).

### Participants

Adult men and women aged 18 years and older and diagnosed with postsurgical, autoimmune, genetic, or idiopathic chronic hypoparathyroidism were eligible to participate in the trial (23). Diagnosis of hypoparathyroidism was established based on hypocalcemia in the setting of inappropriately low serum PTH levels, and chronic hypoparathyroidism was defined as lasting at least 26 weeks. Participants were required to have a body mass index ranging from 17 to 40 kg/m^2^ and to be on stable doses of active vitamin D and calcium for at least 12 weeks prior to screening. Detailed inclusion and exclusion criteria have been previously published (23).

### Trial protocol

Study procedures were performed in accordance with the PaTH Forward clinical trial protocol and have been described in detail elsewhere (23). Active vitamin D and elemental calcium doses were optimized for participants before trial initiation. For the 4-week double blinded period of PaTH Forward, participants were randomized into 3 different palopegteriparatide treatment groups (15, 18, or 21 µg daily dose) or to the placebo group (with randomized doses mimicking the active treatment groups). The drug was administered subcutaneously using prefilled pens. During the blinded portion of the trial, active vitamin D and therapeutic doses of calcium were decreased and/or discontinued in accordance with the conventional therapy adjustment predefined in the trial protocol.

In the open-label extension period of the same trial, participants (including those initially randomized to placebo during the first 4 weeks of the trial) were assigned to a palopegteriparatide dose (15, 18, or 21 µg daily dose) based on their need for active vitamin D at week 4. Palopegteriparatide doses were then titrated for each participant at subsequent trial visits based on serum calcium and symptoms. Active vitamin D and calcium doses were gradually withdrawn. During the extension period, palopegteriparatide doses ranged from 6 to 60 µg/day, with titration based on serum calcium levels.

### Patient-reported outcome measures

Patient-reported outcome (PRO) measures were included in the trial protocol as exploratory endpoints. PRO measures were administered to participants at baseline (during the screening period) and prior to their first dose of the study drug and at weeks 4, 12, 26, 58, 110, 162, and 214 of the trial.

General health-related QoL was assessed using the 36-item Short Form Health Survey, version 2 (SF-36v2) acute version, a validated, self-report measure of perceived health status over the previous week (26). This measure was not administered at week 12. The SF-36v2 includes 36 items that are scored into 8 subscales reflecting different domains of general health and well-being, including physical functioning, role-physical, bodily pain, general health, vitality, social functioning, role-emotional, and mental health. Subscale scores are combined to form the Physical Component Summary (PCS) and Mental Component Summary (MCS) scores. The component and subscale measures are scored and transformed into *T*-scores ranging from 0 to 100, with higher scores indicating better health status. Scoring is norm-based with a mean value of 50 and a SD of 10 in the general US population. For group-level data, scores within 0.3 SD or 3 T-score points from the mean (47-53) are considered within the average US population range.

The Hypoparathyroidism Patient Experience Scales (HPES) are 2 independent, psychometrically validated PRO measures that were developed to assess disease-specific symptoms (HPES-Symptom) and impacts related to hypoparathyroidism (HPES-Impact) in adults (27-29). The HPES-Symptom is a 17-item measure that assesses symptoms of hypoparathyroidism and consists of a total score and 2 domain scores for physical symptoms and cognitive symptoms. Response options assess symptom frequency and are based on a 5-point Likert-type scale, ranging from very often/always (scored as 4) to never (scored as 0). The HPES-Impact is a 26-item measure that assesses impacts on daily functioning and well-being related to hypoparathyroidism and includes a total score and 4 domain scores for physical functioning, daily life, psychological well-being, and social life and relationships. A 5-point, Likert-type scale is used for response options, which range from extremely (scored as 4) to not at all (scored as 0). For both the HPES-Symptom and the HPES-Impact, individual item scores are added to calculate raw total scores and raw domain scores. Raw total and domain scores are then transformed linearly to scale scores that range from 0 to 100, with higher scores indicating greater symptom frequency or greater impact.

### Statistical analysis

Descriptive statistics were used to summarize background characteristics and key outcome variables. In general, continuous variables were summarized using mean, median, SD/standard error (SE), minimum/maximum values, and number of participants. All PRO summary, subscale, and domain measures were analyzed as continuous scale scores. Categorical variables were summarized by the number and percentage of participants. Only observed values were included in the analyses. All statistical tests were 2-sided, and statistical significance was set at α = .05.

Analysis of PRO outcomes for the extension period was conducted based on assessments at baseline and weeks 4, 12, 26, 58, and 110 for all participants who received at least 1 dose of palopegteriparatide. Descriptive analyses of changes from baseline to weeks 12, 26, 58, and 110 were conducted for all measures, including summary and domain scores. The one-sample Student's *t* test was used to assess the significance of the change in PRO scores from baseline.

In a post hoc analysis, Mixed Models for Repeated Measures (MMRM) methodology was used to investigate correlations between baseline demographics and clinical characteristics and the changes from baseline in PRO outcomes measures on 4 selected key HPES domains, including the HPES-Impact physical functioning domain, HPES-Impact daily life domain, HPES-Symptom cognitive domain, and HPES-Symptom physical domain, and the SF-36v2 physical functioning subscale. For each HPES domain and the SF-36v2 subscale, the MMRM model included change from baseline in the PRO measure as the dependent variable, the demographics and baseline characteristics including age, sex, menopausal status (pre- vs postmenopausal status), weight, etiology (surgical, other), duration of hypoparathyroidism, prior PTH therapy (yes, no), daily pill burden for conventional therapy at baseline, elemental calcium dose, active vitamin D dose, and geographic region (North America, other), baseline PRO value of the same domain, and assessment time (weeks 12, 26, 58, and 110) as fixed effects, and participant as a random effect.

## Results

### Participant characteristics

Participant demographic and background characteristics of the PaTH Forward trial participants are presented in Table 1. A total of 59 participants were originally enrolled in the trial, 2 discontinued during the extension period, and 57 completed the week 110 visit. The mean participant age was 49.8 years (SD, 12.1; range, 25-76). The majority of participants were female (81%, n = 48), and most participants identified as White (92%, n = 54). Approximately two-thirds of respondents resided in North America (64%, n = 38), and the remainder resided in Europe (36%, n = 21). The most frequently reported etiology of hypoparathyroidism was neck surgery (80%, n = 47), followed by idiopathic disease (19%, n = 11), and autoimmune disease (2%, n = 1). Participants with postsurgical hypothyroidism were well-controlled with levothyroxine as demonstrated by mean thyrotropin (TSH) within the normal range.

**Table 1.** Baseline demographic and clinical characteristics of participants in the PaTH Forward trial

	Participants (n = 59)
**Age** (years), mean (SD)	49.8 (12.1)
Median	48.4
Range	25-76
**Age group** (years), n (%)	
<30	3 (5)
30 to <50	29 (49)
50 to <65	22 (37)
≥65	5 (9)
**Sex**, n (%)	
Female	48 (81)
Male	11 (19)
**Weight** (kg), mean (SD)	76.3 (16.9)
**Race**, n (%)	
White	54 (92)
Other	5 (8)
**Geographical region**, n (%)	
North America	38 (64)
Europe	21 (36)
**Etiology of hypoparathyroidism**, n (%)	
Neck surgery	47 (80)
Idiopathic disease	11 (19)
Autoimmune disease	1 (2)
**Duration of hypoparathyroidism** (years)	
Mean (SD)	11.9 (9.2)
Range	1-39
**Conventional therapy at baseline** *^a^*	
Calcium, n (%)	54 (100)
TDD (mg), mean (SD)	1909 (1271)
≤1000 mg TDD, n (%)	10 (19)
≤2000 mg TDD, n (%)	34 (63)
Calcitriol (active vitamin D), n (%)	31 (78)
TDD (µg), mean (SD)	0.8 (0.5)
Alfacalcidol (active vitamin D), n (%)	13 (22)
TDD (µg), mean (SD)	2.15 (1.08)
**PTH therapy within 6 months of screening**,*^b^* n (%) yes	12 (20)

Percentages may not add up to 100 due to rounding.

Abbreviations: PTH, parathyroid hormone; TDD, total daily dose.

^*a*^Baseline data available for calcium (n = 54), calcitriol or alfacalcidol (n = 59).

^*b*^Includes PTH(1-84), PTH(1-34), or other N-terminal fragments or analogues of PTH or PTH-related protein.

The mean total daily dose (TDD) of elemental calcium at baseline was 1909 mg (SD, 1271). There were 12 participants (20%) who indicated that they received PTH therapy within 6 months prior to screening for the trial. Among these respondents, the average time between discontinuing prior PTH therapy and starting the trial treatment was 4.1 months (SD, 1.1; range, 2.5-6.3).

### Hypoparathyroidism Patient Experience Scales

All participants completed the HPES-Symptom and HPES-Impact measures at baseline (100%, n = 59), and compliance remained high, with 75% of participants completing the HPES at week 12 (n = 44), 81% at week 26 (n = 48), 95% at week 58 (n = 56), and 97% at week 110 (n = 57).

**Figure 1.**
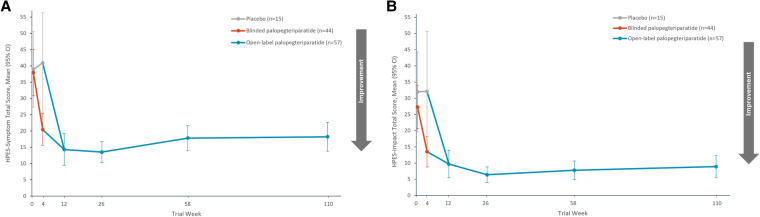
Palopegteriparatide treatment improves mean HPES-Symptom and Impact total scores. A) Mean HPES-Symptom total scores from baseline through week 110 of the PaTH Forward trial; B) Mean HPES-Impact total scores from baseline through week 110 of the PaTH Forward trial. The total number of participants with HPES data varied at some time points: n = 44 at week 12, n = 48 at week 26, and n = 56 at week 58. Error bars indicate 95% CI. Higher scores indicate more frequent symptoms and greater negative impact. Abbreviation: HPES, Hypoparathyroidism Patient Experience Scale.

Compared with placebo, treatment with palopegteriparatide during the 4-week blinded period resulted in a statistically significant improvement in both HPES-Symptom and Impact total and domain scores (*P* < .01). This immediate improvement was evident at the first time point measured. The initiation of open-label palopegteriparatide treatment at week 4 in participants randomized to placebo at baseline led to a rapid improvement (decrease) in HPES scores similar to the group initially randomized to active treatment (Fig. 1). Overall, the HPES-Symptom total scale score mean improved significantly from baseline to week 12, and this improvement was maintained through weeks 26, 58, and 110 (Fig. 1A). At baseline, the average HPES-Symptom total score was 38.2 (SE, 2.9), and it decreased significantly to 13.5 (SE, 1.6; *P* < .0001) at week 26, an improvement of 24.0 points (SE, 3.2; *P* < .0001). From baseline to week 58, the average HPES-Symptom total score was improved by 20.5 points (SE, 2.6; *P* < .0001). Significant improvement of 19.7 points (SE, 2.8; *P* < .0001) from baseline was maintained at week 110. Improvements in both the physical and cognitive symptom domain scores over the course of the extension period were similar (Table S1) (30).

The HPES-Impact total scale score and domain scores also improved significantly from baseline to week 12, and significant improvements in impacts from baseline were maintained through week 110 (Fig. 1B). At baseline, the HPES-Impact total scale score mean was 28.4 (SE, 2.9), and it improved significantly by 15.3 points (SE, 2.9; *P* < .0001) at week 12. The HPES-Impact total score continued to be significantly improved from baseline by 22.4 points (SE, 3.1; *P* < .0001) at week 26 and by 20.7 points (SE, 2.8; *P* < .0001) at week 58. A significant improvement in the mean HPES-Impact total score of 19.0 points (SE, 2.8; *P* < .0001) from baseline persisted at week 110. Similar patterns of improvement in impacts on functioning and well-being were observed in all of the HPES domain measures (Table S1) (30).

### General health-related quality of life

**Figure 2.**
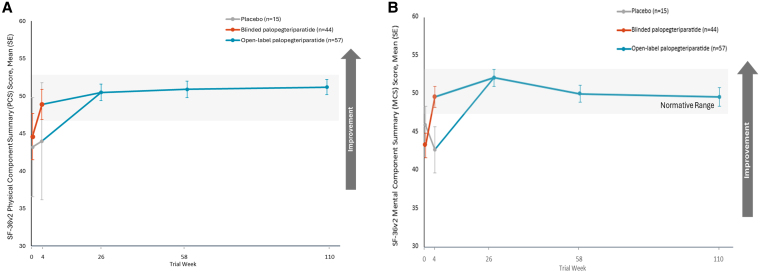
Palopegteriparatide treatment improves mean SF-36v2 physical component summary (PCS) and mental component summary (MCS) scores. A) Mean SF-36v2 PCS scores from baseline through week 110 of the PaTH Forward trial; B) Mean SF-36v2 MCS scores from baseline through week 110 of the PaTH Forward trial. The total number of participants with SF-36v2 data varied at some time points: n = 48 at week 26 and n = 56 at week 58. Error bars indicate 95% CI. Higher scores indicate better reported health status. Shaded regions show the normative mean score range for the US (47-53) (26). Abbreviation: SF-36v2, Short Form Health Survey, version 2.

All respondents completed the SF-36v2 measures at baseline (100%, n = 59). The SF-36v2 was completed by 81% of participants at week 26 (n = 48), 95% at week 58 (n = 56), and 97% at week 110 (n = 57). Similar to HPES scores, mean SF-36v2 component summary scores rapidly improved from below normal at baseline into the normative range with palopegteriparatide treatment, and this improvement was sustained over the open-label extension period. The average PCS and MCS scores at baseline were below normative values for the US population, which range from 47 to 53 (26). Both the PCS and MCS mean scale scores increased significantly from baseline to week 26 to values within the normative US population mean range, and these improvements were maintained through weeks 58 and 110 (Fig. 2).

At baseline, the mean PCS score was 44.2 (SE, 1.4), which improved significantly by 6.0 points (SE, 1.1; *P* < .0001) at week 26 and 6.9 points at week 58 (SE, 1.0; *P* < .0001). This durable improvement in the PCS score remained evident at week 110, with an increase of 6.7 points from baseline (SE, 1.2; *P* < .0001). The mean baseline MCS score was 43.9 (SE, 1.4) which likewise improved significantly by 8.2 points (SE, 1.4; *P* < .0001) at week 26 to 52.1, reaching the normative range of 47 to 53 for the US population. Normalization of the mean MCS score was maintained through week 110, with a significant improvement of 5.8 points (SE, 1.4; *P* = .0002). Trends for all of the subscale scores of the SF-36v2 were similar (Table S2) (30).

### Mixed model for repeated measures analyses

**Figure 3.**
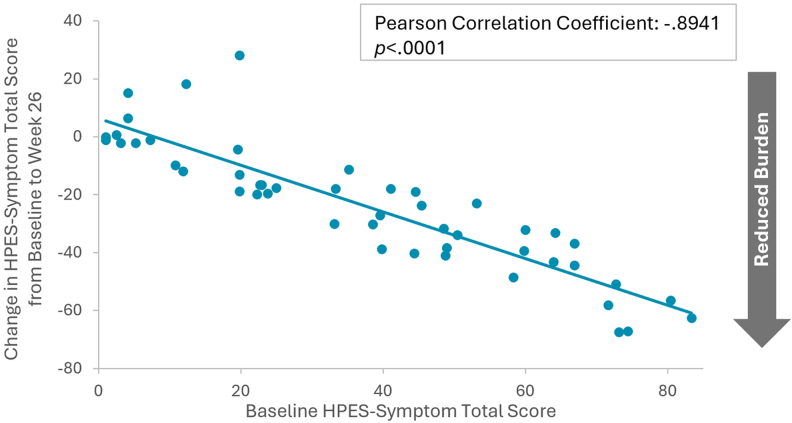
Correlation between HPES-symptom total baseline scores and changes in scores from baseline to week 26 of the PaTH Forward trial, n = 48. Negative change values indicate reduced burden from baseline to week 26. Abbreviation: HPES, Hypoparathyroidism Patient Experience Scales.

**Figure 4.**
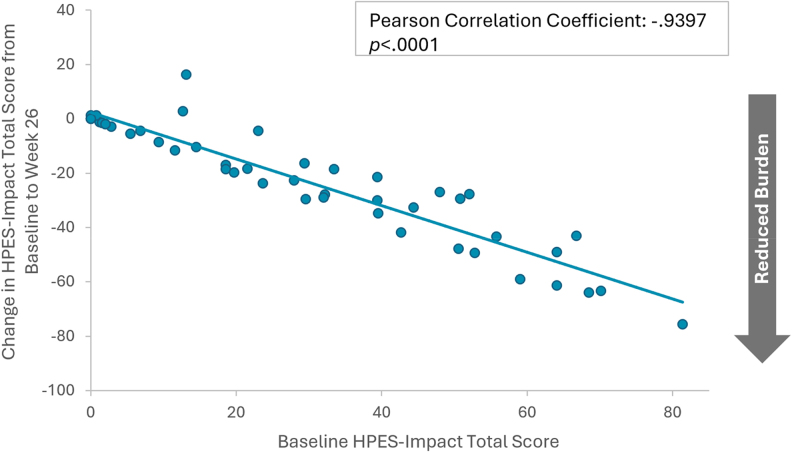
Correlation between HPES-impact total baseline scores and changes in scores from baseline to week 26 of the PaTH Forward trial, n = 48. Negative change values indicate reduced burden from baseline to week 26. Abbreviation: HPES, Hypoparathyroidism Patient Experience Scales.

Results of the post hoc MMRM analyses indicated that improvements in key PRO measures from baseline were generally similar across all baseline demographics and clinical characteristics evaluated (Table S3) (30). The strongest correlate of change in PRO measures over the course of the open-label extension period was baseline scale score. Worse baseline PRO scores (ie, higher HPES scores or lower SF-36v2 scores) were strongly associated with greater improvements in outcomes compared to better baseline PRO scores across all 4 HPES domains and the SF-36v2 physical functioning subscale (all *P* < .0001). Pearson correlations and scatterplots illustrate the relationship between baseline scores and change from baseline to week 26 for the HPES-Symptom total score (Fig. 3) and the HPES-Impact total score (Fig. 4). Correlations between baseline scores and changes from baseline to week 58 and correlations between baseline scores and changes to week 110 were similar.

Across the key HPES and SF-36 measures, greater improvement tended to associate with younger age, postmenopausal status, male sex, surgical etiology, longer duration of hypoparathyroidism, and higher calcium dose at baseline. These variables showed consistent trends across all 5 measures but did not reach statistical significance. Participants who received PTH therapy in the 6 months prior to screening for the trial experienced slightly less improvement in the HPES-Symptom physical and cognitive domains, and the HPES-Impact physical functioning domain than those with no PTH therapy in the 6 months prior to screening.

The relationship between improvement in PROs and calcium homeostasis was examined by a correlation analysis between change in PRO domain scores and change in serum calcium post-baseline. Improvement in symptoms, impacts, well-being, and health-related QoL did not strongly correlate with change in serum calcium (Table S4).

## Discussion

PTH replacement therapy with palopegteriparatide in the PaTH Forward trial resulted in significant and sustained improvements in disease-specific symptoms and impacts on daily functioning and well-being, as well as in general health-related QoL in adults with chronic hypoparathyroidism. Significant improvements in both the HPES and SF-36v2 measures were evident immediately after the initiation of palopegteriparatide during the 4-week blinded period in participants randomized to palopegteriparatide, and after receiving open-label palopegteriparatide for those initially randomized to placebo. Significant improvements from baseline for all HPES and SF-36v2 measures persisted for more than 2 years after the first exposure to palopegteriparatide, demonstrating the robust positive impact of PTH replacement therapy in individuals with chronic hypoparathyroidism. These results expand and reinforce previous analyses of palopegteriparatide that were the first to demonstrate significant improvements in symptoms and impacts on QoL with PTH replacement therapy in double-blind, randomized controlled clinical trials of adults with chronic hypoparathyroidism (23, 24). These results also affirm the importance of disease-specific measures for their ability to assess the full range of important and relevant symptoms and impacts associated with a condition, as well as their sensitivity to change (31).

The findings underscore the importance of PTH replacement therapy to address insufficient PTH and the limitations of conventional therapy. It is noteworthy that the majority of patients achieved meaningful within-patient change for both HPES-Symptom and HPES-Impact through week 110 of the PaTH Forward clinical trial (23, 29). This is particularly important given the high symptom burden and poor QoL experienced among adults with chronic hypoparathyroidism, despite management with conventional therapy, compared to population norms (5, 6, 11, 12).

Hypoparathyroidism symptoms improved with the initiation of palopegteriparatide treatment in the PaTH Forward trial. Specifically, after 2 years of treatment, HPES-Symptom scores were lower than baseline by 19 to 20 points, on average. These group-level changes met or exceeded the recommended threshold range of 15 to 19 points (transformed) to demonstrate meaningful within-patient improvement in the HPES-Symptom measures (29). Additionally, improvements in the HPES-Impact measures after more than 2 years of palopegteriparatide treatment ranged from about 15 to 23 points. These changes also generally met or exceeded the proposed threshold range of 13 to 18 points (transformed) to indicate within-patient meaningful improvement in the HPES-Impact measures (29). Evaluating the HPES group-level changes against within-patient meaningful improvement thresholds represents a more rigorous comparison than using group-level thresholds. In addition to the HPES results, improvements in the SF-36v2 assessments from baseline through week 110 exceeded generally suggested values for indicating group-level minimally important differences (MID) for both the PCS (2 points) and the MCS (3 points), as well as all domain score measures (26).

The strongest and most consistent predictors of improvements in HPES outcomes with palopegteriparatide treatment were baseline HPES scores. Worse baseline PRO scores (eg, higher HPES scores and lower SF-36v2 scores) were associated with significantly greater improvement in scores over the trial extension period for all measures. Other participant baseline characteristics were not generally predictive of treatment-related improvements in symptoms and impacts related to hypoparathyroidism. Improvements in symptoms, impacts on daily functioning and well-being, and health-related QoL were generally similar, regardless of baseline age, sex, hypoparathyroidism etiology and duration, serum calcium, and conventional therapy use. This is consistent with previous research that has found limited associations between general QoL and patient background characteristics, including age, etiology, duration of disease, and clinical measures such as serum calcium, in adults with hypoparathyroidism (12, 13, 32). Slightly less improvement in the HPES-Symptom physical and cognitive domains and HPES-Impact physical functioning domain was reported by participants who received PTH therapy within 6 months of starting the trial than those with no PTH therapy in the 6 months prior to baseline. This suggests that these individuals may have experienced an incremental improvement in quality of life with previous use of short-lived PTH therapies, consistent with the results of a meta-analysis by Puliani et al (33), and then further improvements in hypoparathyroidism-related symptoms and impact with palopegteriparatide. Overall, these findings demonstrate that PTH replacement therapy with palopegteriparatide may offer long-term benefits to symptoms, daily functioning and well-being, and health-related QoL in adults with chronic hypoparathyroidism.

These findings demonstrate that PTH replacement therapy with palopegteriparatide may be particularly beneficial to adults with chronic hypoparathyroidism who experience a high symptom burden and poor QoL despite management with conventional therapy. The mechanisms underlying improvements in participants' well-being and QoL are not fully understood. The prevention of fluctuations in serum calcium levels throughout the day likely plays an important role in ameliorating symptoms and thus improving well-being. It can also be speculated that PTH itself might play a role. PTH is a ligand for PTH1R and PTH2R, which are both expressed in several regions of brain (34-36). PTH replacement therapy with palopegteriparatide provides the physiological effects of endogenous PTH at the receptor and tissue level. Thus, active PTH released from palopegteriparatide may potentially play a direct role in the observed treatment-related improvements in QoL, though the effect of PTH receptor binding in the brain on cognition and well-being warrants further investigation. Recently, it was reported that brain structural changes might be present in individuals with hypoparathyroidism on conventional therapy. A cross-sectional magnetic resonance imaging study demonstrated a correlation between a longer duration of hypoparathyroidism and smaller hippocampal volume, as well as a negative correlation between hippocampal volume and self-reported severity of slow or confused thinking (“brain fog”) (3). It remains to be seen if PTH replacement therapy with palopegteriparatide can reverse these structural brain changes. Ongoing research will explore the longer-term implications of treatment with palopegteriparatide for improvements in symptoms and impacts on functioning and well-being specific to hypoparathyroidism, as well as the potential for reductions in treatment burden and risks of serious long-term complications associated with conventional therapy (1, 6, 8, 13).

Potential limitations of this analysis should be considered. One limitation is that the interval of direct comparison to the placebo group in the blinded period was relatively short (4 weeks) and most data were drawn from an open-label clinical trial extension period, without a placebo-control group for comparison at all time points. Nevertheless, the findings were consistent with previous rigorous research based on randomized, double-blind, placebo-controlled trials of palopegteriparatide (23, 24). The sample size was relatively small and prevented a comparison between outcomes in surgical and nonsurgical patients, but it was appropriate for a clinical trial of a rare condition and was generally representative of adults with hypoparathyroidism. The racial and ethnic diversity of the study sample, however, was limited. Normative data for the SF-36v2 were not available for all participating countries in the trial, and the US default normative data were used (26), potentially limiting the interpretation of the normative scoring due to cultural differences in self-reported measures among participating countries (37).

In conclusion, PTH replacement therapy with palopegteriparatide in adults with chronic hypoparathyroidism demonstrated significant and sustained improvement in symptoms and impacts on functioning and well-being associated with hypoparathyroidism and general QoL.

## Data Availability

Some or all datasets generated during and/or analyzed during the current study are not publicly available but are available from the corresponding author on reasonable request.

## Abbreviations

HPES: Hypoparathyroidism Patient Experience Scale
MCS: Mental Component Summary
MMRM: Mixed Models for Repeated Measures
PCS: Physical Component Summary
PTH: parathyroid hormone
PRO: patient-reported outcome
QoL: quality of life
SF-36v2: 36-item Short Form Health Survey, version 2
