# Correction to: “Patient-reported outcomes in palopegteriparatide-treated adults with hypoparathyroidism: PaTH Forward trial extension”

**DOI:** 10.1210/clinem/dgag021

**Published:** 2026-01-28

In the above-mentioned article by Rubin M, Palermo A, Vokes T, Khan AA, Schwarz P, Cetani F, Pagotto U, Tsourdi E, Sikjaer T, Pfeiffer KM, Brod M, McLeod LD, Zhao C, Noori W, Shu AD, and Smith AR (*J Clin Endo Metab*. 2025; doi: 10.1210/clinem/dgaf653), there was an error in the citation for reference 30.

In the originally published article, in the References section, reference 30 read “Placeholder. Supplementary Material Data Repository. Doi: 10.6084/m9.figshare.30888056.” The word “Placeholder” has been replaced with the author information “Rubin M, Palermo A, Vokes T, *et al*.” The title of the reference has been updated to reflect the title of the webpage cited, changed from “Supplementary Material Data Repository” to “JCEM Rubin et al Supplemental Material 2025.” The source website “Figshare” has been added in the citation, and the DOI has been changed from “10.6084/m9.figshare.30888056” to “10.6084/m9.figshare.30888056.v1.” In the corrected article, reference 30 now reads “Rubin M, Palermo A, Vokes T, *et al*. JCEM Rubin et al Supplemental Material 2025. *Figshare*. 2025. doi:10.6084/m9.figshare.30888056.v1.”

The article has been corrected online.

doi: 10.1210/clinem/dgaf653

